# Use of Photodynamic Therapy Associated with Antimicrobial Peptides for Bacterial Control: A Systematic Review and Meta-Analysis

**DOI:** 10.3390/ijms23063226

**Published:** 2022-03-17

**Authors:** Luana Mendonça Dias, Túlio Morandin Ferrisse, Karine Sousa Medeiros, Eduardo Maffud Cilli, Ana Claudia Pavarina

**Affiliations:** 1 Laboratory of Applied Microbiology, Department of Dental Materials and Prosthodontics, School of Dentistry, São Paulo State University (UNESP), Araraquara 14801-903, Brazil; luana.dias@unesp.br (L.M.D.); tulio.m.ferrisse@unesp.br (T.M.F.); karine_maraujo@hotmail.com (K.S.M.); 2 Department of Biochemistry and Organic Chemistry, Institute of Chemistry, São Paulo State University (UNESP), Araraquara 14800-900, Brazil; eduardo.cilli@unesp.br

**Keywords:** antimicrobial peptides, photochemotherapy, anti-infective agents

## Abstract

Considering the challenges related to antimicrobial resistance, other strategies for controlling infections have been suggested, such as antimicrobial photodynamic therapy (aPDT) and antimicrobial peptides (AMP). This study aims to perform a systematic review and meta-analysis to obtain evidence on the antimicrobial effectiveness of aPDT associated with AMP and establish in vitro knowledge on this topic for further study designs. The PubMed, Scopus, Web of Science, Science Direct, Scielo, and Cochrane Library databases were searched. Two independent and calibrated researchers (Kappa = 0.88) performed all the systematic steps according to the Preferred Reporting Items for Systematic Reviews and Meta-Analyses (PRISMA). The odds ratio (OR) was used as the effect measure. The Peto method was used to perform the meta-analysis due to the sparse data. Twenty studies were included in the present review. The result was significant (OR = 0.14/*p* = 0.0235/I-squared = 0%), showing better outcomes of aPDT associated with peptides than those of aPDT alone for controlling the microbial load. Only 20% of the studies included evaluated this approach in a biofilm culture. Combined treatment with aPDT and AMP highly increased the ability of microbial reduction of Gram-positive and Gram-negative bacteria. However, additional blind studies are required to evaluate the efficacy of this therapy on microbial biofilms.

## 1. Introduction

Antimicrobial resistance to conventional drugs has resulted in high global rates of recurrent invasive infections, facilitating disease progression and reducing the likelihood of effective treatments [1]. In 2020, the World Health Organization warned about the appearance of strains increasingly resistant and difficult to control. The indiscriminate use of antimicrobial drugs is facilitated by inadequate medical prescriptions and substandard medications [2].

Considering the challenges related to antimicrobial resistance, other strategies for controlling infections have been suggested [3,4,5,6]. Antimicrobial photodynamic therapy (aPDT) has been used to inactivate microorganisms and treat infections [3,4,5,6]. aPDT involves the application of a photosensitizing agent (PS), an LED source corresponding to the absorption band of the PS, and the presence of oxygen. This therapy has several advantages in the treatment of infections from microorganisms, such as the wide spectrum of action and a low mutagenic potential in exposed cells [6].

When comparing aPDT with other therapies, it has the advantage of local PS application, restricting the treatment to the area of interest, thus preventing systemic side effects. There is also an immediate onset of action and elimination of virulence factors secreted by resistant microorganisms [7]. Lastly, the literature did not report the development of bacteria and fungi resistance to aPDT [4,8].

Studies have shown that microbial biofilms reduce the susceptibility to aPDT compared to planktonic cultures [4]. Considering the protection endowed by the extracellular matrix (ECM), it is difficult for the PS to penetrate the deeper layers of the microbial biofilm, impairing aPDT activity [9]. To overcome this limitation, aPDT associated with enzymes or antifungal agents was more effective for microbial inactivation than aPDT alone [5,9]. Additionally, antimicrobial peptides (AMP) have been used alone [10,11], combined with aPDT [12,13], or by conjugating a PS to the AMP molecule [14,15,16,17,18,19,20,21,22,23,24,25,26,27,28,29,30,31], presenting satisfactory results in pathogenic microorganism inactivation.

AMP are molecules expressed by all living organisms and responsible for the innate defense system against pathogen infection, including viruses, bacteria, fungi, and parasites [32]. AMP are oligopeptides with up to 50 amino acids with a broad spectrum of action against microorganisms [33,34]. This new class of compounds has boosted science for new methodologies for synthesizing, isolating, purifying, analyzing, and quantifying peptides [35]. The presence of cationic residues (Arg and Lys) in AMP promotes a positive liquid charge for this structure, resulting in the interaction with the negative cell membrane of the target organism, such as bacteria [35]. Another important aspect of the construction of the AMP amphipathic structure is the high fraction of hydrophobic amino acids (>50%) [36], which is vital for membrane penetration. The biological activity of AMP is closely related to their structure, and these could be classified as α-helix, β-sheet, extended peptides, and both α-helix and β-sheet peptides [37], with the first two appearing more frequently [38]. Although the molecular target of some peptides is inside the cell, as non-membrane disruptive AMP [39], most peptides interact with the anionic components of the membranes of microorganisms and damage this structure [32].

The literature has described the association of AMP and aPDT to explore the best properties of both treatments, increasing the effectiveness and decreasing the time of application [12,13]. AMP can form pores in cell membranes and present biofilm activity [11], which leads to the penetration of the PS into the membrane, facilitating the inactivation of structures through LED photoexcitation [12]. Other advantages of association treatments are reduced effective dose, minimized toxicity potential, and reduced treatment costs [12,40].

To elucidate the antimicrobial efficacy of aPDT associated with AMP, this study performed a systematic review and a meta-analysis by searching the existing literature. The data synthesis provided in the present study establishes in vitro knowledge on this topic for different study designs.

## 2. Results

### 2.1. Search Results

**Figure 1:**
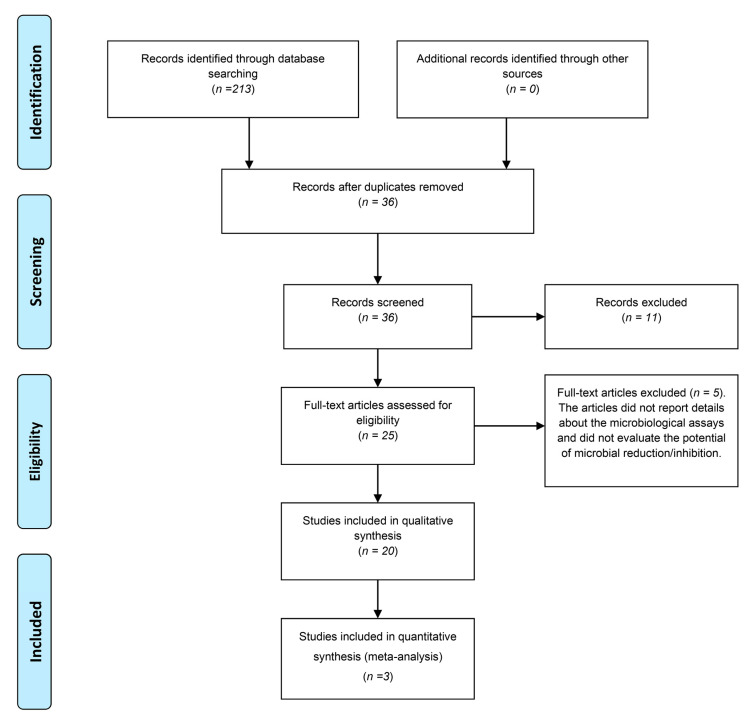
Flowchart based on the PRISMA statement.

The flowchart in Figure 1 shows the process of article selection. The preliminary electronic search yielded 213 articles. After excluding duplicates, 36 studies remained. The titles and abstracts were read, and no article was excluded. After evaluating the abstracts, 25 studies were considered for a full-text evaluation. Then, 5 articles were excluded because they did not report details about the predetermined microbiological assays.

### 2.2. Synthesis of Results

The results of the systematic review show that all articles had an in vitro experimental design and 3 of them were both in vitro and in vivo experimental studies [28,30,31]. Moreover, of the 20 articles analyzed, 18 performed the therapy with a portion of the PS redirected to AMP and only 2 studies performed the therapy combined with AMP [12,13]. The shortest and longest irradiation times were 30 s [14] and 20 h [21,22], respectively. The most commonly used PS were chlorin e6 [12,13,24,28,30,31] and porphyrin [14,16,17,19,21,22,25,26]. Additionally, the most frequently used microorganism in the assay was *Staphylococcus aureus* [12,14,15,17,18,19,20,21,22,24,26,27,29,30,31], followed by *Escherichia coli* [12,14,16,17,18,19,20,21,22,26,27,30,31]. Most of the studies analyzed evaluated the microorganisms in suspension (planktonic culture) and only 4 evaluated the therapy in a biofilm culture [12,24,28,31] (Table 1).

### 2.3. Risk of Bias Assessments for In Vitro Studies

The criteria from the OHAT Rob tool were applied to all articles included in the systematic review. The most frequent biases regarded blinding procedures. Moreover, the problem with internal validity was the lack of methodological details in the statical analyses and the performance of treatments only in microorganism suspensions (Table 2).

### 2.4. Meta-Analysis

The meta-analysis was performed only in 3 studies [13,15,23]. The reduced number of studies included in the quantitative analysis is due to the lack of data (e.g., sample size) and the absence of a study group evaluating only aPDT application. The experimental group included microorganisms treated with aPDT associated with peptides (aPDT + AMP), while the control group included microorganisms treated only with aPDT (aPDT). The microbial load was the outcome evaluated in the meta-analysis.

**Figure 2:**
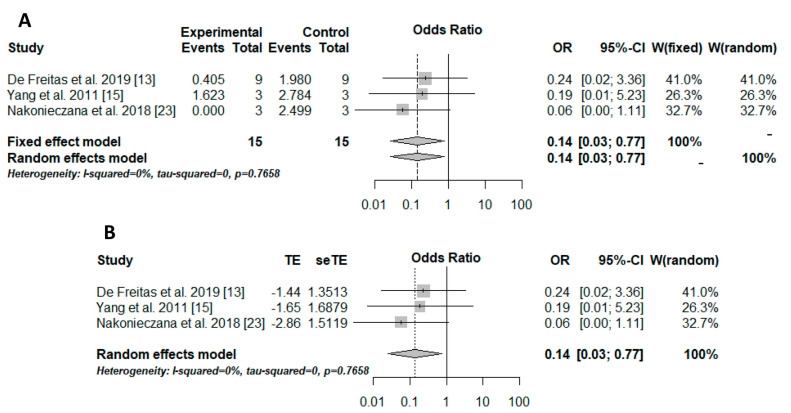
Ilustration of the results of the quantitative analysis. The experimental group (positive events) included microorganisms that received the association therapy (aPDT + AMP), while the control group included microorganisms that received only aPDT. (**A**) results of the meta-analysis illustrated in a forest plot. OR: odds ratio; CI: confidence interval; W: weight, [13,15,23]. (**B**) trim-and-fill method results illustrated in a forest plot. TE: estimated mean; seTE: estimated standard deviation; OR: odds ratio; CI: confidence interval; W: weight, [13,15,23].

The Peto method was used to perform the meta-analysis due to the sparse data. The results were transformed into odds, and, therefore, the odds ratio (OR) was used as the effect measure. The result was significant (OR = 0.14/*p* = 0.0235/I-squared = 0%), showing better outcomes for aPDT associated with peptides than those for aPDT alone for controlling the microbial load (Figure 2A). Moreover, small-study effects in the meta-analysis and consequently publication and meta-analysis biases were verified with the trim-and-fill method. However, there were no biases (Figure 2B).

## 3. Discussion

The exposure of bacteria to conventional antimicrobial agents often leads to a selection of strains that are more resistant to many of these drugs [1,41]. To inactivate the microorganism and overcome the progress of the infection, alternative strategies may be suggested, such as using aPDT and AMP [12,13,14,15,16,17,18,19,20,21,22,23,24,25,26,27,28,29,30,31]. This study performed a systematic review and a meta-analysis to elucidate the antimicrobial efficacy of aPDT associated with AMP by searching the existing literature. To achieve better results for antimicrobial inactivation, all variable situations of these two treatments require a precise control [6,7]. For instance, irradiation time and type of photosensitizer are among the main variables associated with the treatment success of aPDT [7]. Moreover, in the clinical application of this therapy, success is determined based on infection remission and consequently the restoration of site function [5,7]. Additionally, in AMP treatments, peptide size, positive charge, conformation, and stability are characteristics related to antimicrobial achievement [35]. The association of AMP with aPDT has been described in the literature to explore the best properties of both treatments, increasing the effectiveness against microorganisms.

To improve the efficacy of aPDT in reducing the microbial load, the N-terminal of AMP has been chosen to conjugate with the photosensitizer molecule [14,15,16,17,18,19,20,21,22,23,24,25,26,27,28,29,30,31]. This approach may increase water solubility and facilitate the penetration of the component into bacterial membranes through pore formation [26]. Furthermore, AMP-like compounds represent a promising alternative to broad-spectrum antibiofilm agents, with synergistic activities against persistent infections caused especially by biofilm formation [35]. Among the studies evaluated, 80% used the conjugated therapy approach [14,15,16,17,18,19,20,21,22,23,24,25,26,27,28,29,30,31].

aPDT and AMP may also be associated by combining individual treatments. Two studies included in the present systematic review investigated this approach [12,13]. The success of the combined therapy can be attributed to the possibility of targeting different cell compartments to increase the damage to target cells. It is also possible to extend the spectrum of action of the therapeutic response [42]. Additionally, the meta-analysis results strengthen the findings supporting the combined therapy (aPDT + AMPs) when compared with a single treatment (aPDT). Combining two or more antimicrobial therapies with different action mechanisms can decrease therapeutic failure due to the reduced likelihood of microorganisms presenting antimicrobial resistance and tolerance to both treatments simultaneously [43,44].

The association of aPDT and AMPs resulted in microbial load reduction by 100% for *Staphylococcus aureus* [12,15,26,30], *Pseudomonas aeruginosa* [28], and *Enterococcus faecium* [12]. A high rate of microbial reduction (>90%) was also found for *Acinetobacter baumannii* [12,20], *Escherichia coli* [16,18,20,27,30], *Staphylococcus epidermidis* [18], and *Enterococcus faecalis* [13]. Lastly, microbial reductions between 50% and 90% were found for *Mycobacterium smegmatis* [25], *Salmonella enteric* [25], and *Klebsiella pneumoniae* [25]. *S. aureus*, followed by *E. coli*, were the bacteria most frequently evaluated in the articles included. This preference can be attributed to the pathogenicity and consequently a high degree of infection of these microorganisms. These microorganisms are also recognized as a major threat to human and animal health [30,31]. For instance, *E. coli* is known to inhabit mainly the lower intestinal tract of humans, causing intestinal and urinary infections. In addition, the increased pathogenicity of *E. coli* can affect the central nervous system of the host, causing inflammation and meninges [45]. Gram-positive *S. aureus* bacteria are associated with persistent nosocomial colonization in up to 25% of the healthy adult population, potentially causing bacteremia and subcultural abscesses [46]. Studies have shown that persistent chronic infections caused by *S. aureus* are related to bacterial growth in a biofilm model and may be fixed in bone and heart valves or implanted materials [47].

A biofilm is defined as a sessile microbial community with cells adhered to a surface and incorporated by a polymeric extracellular matrix (ECM) [48]. The ECM composition varies among strains and may contain host factors, polysaccharides, proteins, and extracellular DNA (eDNA) [48,49,50]. These components provide immune protection and antibiotic resistance and tolerance to microorganisms growing inside the biofilm [51]. Reproducing this culture is relevant because of the potential microbial growth in any humid biotic and abiotic surface [52], which makes it more representative than suspension cultures. Nevertheless, among the articles evaluated in the present study, only 20% performed the research in a biofilm culture. This is because suspension cultures are commonly used in preliminary studies, selecting the better approaches for antimicrobial treatment.

The AMPs presented different action mechanisms against planktonic cells and biofilms. The main action mechanisms in planktonic cultures are cytoplasmic membrane effects, cell envelope targets, and intracellular targets [36]. Most of the studies included in the present systematic review used AMPs with membrane effects [12,13,14,15,16,17,18,19,20,21,23,24,26,27,28,29,30,31]. This preference occurs because PS entry is facilitated after the disruption of the membrane surface of microorganism cells. Additionally, the cationic charge in the PS + AMP association increases the possibility of high PS concentration available to enter the cells [36]. Only two studies evaluated AMPs related to cell envelope targets [22,25]. All articles that evaluated the efficacy of AMPs on biofilms in their study design used AMPs with cytoplasmic membrane effects (maturation phase of a biofilm) [13,24,28,31]. In contrast, the scientific literature describes three additional action mechanisms against biofilms, namely the block attachment of cells (attachment phase of a biofilm), matrix disruption (biofilm development), and cell dispersal (dispersal phase of a biofilm) [36]. It has been suggested that increasing membrane permeability would be the most suitable action mechanism of AMPs against biofilm formation. This would facilitate the entry of antibiotics into cells and access to specific intracellular targets [53].

In some cases, AMPs did not present good anti-biofilm responses because it was difficult to inhibit biofilm formation. This can be explained by the minimization of the process related to microorganism adhesion and differences in the AMP amino acid sequence in peptides [13]. The literature reports that a microbial biofilm is more difficult to eliminate with aPDT than planktonic cultures [4]. The extracellular matrix may have an important part in protecting a biofilm against aPDT [3]. This structure complicates the penetration of PS in the existing multilayer of this model, which consequently interferes with the production of ROS and cellular lysis [54]. To overcome this limitation, the association of aPDT with AMPs should be further investigated in biofilm cultures.

In the present systematic review, the most frequently used PS were chlorin e6 [12,13,28,30,31], followed by porphyrins [14,16,17,19,20,21,22,25]. First-generation PS (porphyrin derivatives) have been replaced with second-generation PS (chlorins and phthalocyanines). Chlorins are reduced hydrophilic porphyrins with a strong absorption band in the red region of the spectrum (540–700 nm), resulting in a high therapeutic response even at lower PS concentrations [55]. Considering the action of chlorin in this absorption band, the light penetrates deeper into the tissue [56]. Additional advantages of using chlorins are the shorter photosensitization period, higher quantum yield in singlet oxygen production, and more favorable light absorption characteristics than first-generation PS [55].

The therapeutic light doses of the studies analyzed ranged from 390 to 750 nm. The optimal condition for the photodynamic therapy is the length of light ranging from 400 to 800 nm, known as the therapeutic window, in which there is maximum tissue light transmittance, representing an advantage for the treatment of infections. Above 800 nm, radiation is absorbed by water, which restricts the wavelength to this upper limit [57]. Wavelength radiation lower than 400 nm undergoes greater scattering. The presence of endogenous chromophores in the tissues, which absorb at shorter wavelengths, mainly hemoglobin, reduces light penetration [58].

All articles included in the systematic review have failed to use blinding. This approach is important because it can eliminate biases related to effect size estimates. Thus, the magnitude of the effect remains accurate, the observational bias may be eliminated, and consequently, the results will be more reliable [59]. For potential threats related to internal validity, details on the statistical approaches were considered, but all articles have also failed in this item. Typically, power analysis and sample size estimations are crucial points for rejecting and accepting the null hypothesis [60]. Data normality and homoscedasticity verification are essential steps for making a correct inference [61]. In the present systematic review, only three articles were included in the meta-analysis. Therefore, the results should be interpreted with caution. The main reason for such a small number of studies meta-analyzed was the absence of sample size data reported in the articles.

In short, further studies should be developed evaluating the association between AMPs and aPDT against microorganisms in a biofilm, in addition to blind studies and using AMPs with different action mechanisms. Moreover, only bacterial species were evaluated in the studies analyzed. Therefore, further research using this therapeutic approach against other microorganisms (e.g., fungal species, parasites, and viruses) would be highly recommended.

## 4. Materials and Methods

### 4.1. Protocol and Registration

The present systematic review was performed according to the Preferred Reporting Items for Systematic Reviews (PRISMA) statement [62]. The present study was registered in the Open Science Framework (OSF) (registration doi:10.17605/OSF.IO/2BWDH).

### 4.2. Data Extraction and Research Question

The research question was based on the PICO strategy for systematic exploratory reviews, where P = microorganism, I = aPDT combined with antimicrobial peptides (dual therapy) or aPDT conjugated with peptides, C = isolated therapy (aPDT), and O = reduction in microbial load. The present study aimed to answer the following focused questions: “Does the association between aPDT and AMPs increase the effectiveness of the therapy in reducing the microbial load”? Further data on the name of the first author, the date of publication, study design, peptides used, the sample size, the photosensitizer, the wavelength, the irradiation time, and the microorganism evaluated were extracted from the articles included in this systematic review according to the eligibility criteria.

### 4.3. Eligibility Criteria

The inclusion criteria for this systematic review were the use of aPDT associated with AMPs to reduce the microbial load, including the combined therapy, or PS conjugated with peptides. There were no restrictions on study design (e.g., inclusion of in vitro and in vivo studies, observational human studies, and randomized clinical trials), language, and microorganisms. The exclusion criteria were review articles, case reports, other modalities of treatment using AMPs, and aPDT combined with other modalities of treatment.

### 4.4. Search Strategy

Two independent examiners (L.M.D and T.M.F) were calibrated to select the articles. Thus, the independent examiners conducted an electronic search in PubMed, Web of Science, Scopus, Scielo, Lilacs, and Cochrane Library databases. The search terms were “antimicrobial peptide” and “antimicrobial photodynamic therapy”. A manual search was also performed in other relevant journals in the field of photodynamic therapy and at ClinicalTrials.gov. Based on the titles and abstracts, the same two independent examiners selected and classified the articles as included in or excluded from the review (Kappa score = 0.88). The Rayyan for Systematic Reviews™ software was used to delete duplicate articles [63]. The data were extracted from the articles selected after concluding the eligibility step (Kappa score = 0.87). The studies were analyzed and discussed. Any disagreement during the process was solved by reaching a consensus before proceeding to the next steps.

### 4.5. Meta-Analysis and Quantitative Approaches

The meta-analysis was performed with the R software (version 3.6.3) at α = 0.05. The viability of microbial cells (frequency of positive cells) was the outcome used in the meta-analysis. The experimental group included aPDT associated with AMPs (aPDT + AMPs), while the control group included only aPDT application (aPDT). The random-effects model and the Peto method (presence of sparse data) were used to perform the meta-analysis. The odds ratio was the effect measure selected to perform the quantitative analysis of the binary outcome. To detect the publication bias related to the small-study effect in the meta-analysis, the trim-and-fill method was performed. A high level of heterogeneity was considered for I-squared > 50%.

## 5. Conclusions

Combined treatment with aPDT and AMPs is effective because it increases the ability of microbial reduction for Gram-positive and Gram-negative bacteria. However, additional blind studies are required to evaluate the efficiency of the association between AMPs and aPDT against microorganisms in a biofilm, in addition to blind studies and using AMPs with different action mechanisms. Therefore, further research using this therapeutic approach against other microorganisms would be highly recommended.

## Figures and Tables

**Table 1:** Summary of the characteristics of the studies included.

Study (Year)	Study Design	Peptide	Irradiation Time	Wavelength	Photosensitizer	Microorganism	Culture Type	Sample Size	Outcomes
Bourré et al. 2010 [14]	In vitro	Tat	30, 43, 60, and 120 s	410 nm	Tetracks (phenol) and porphyrin	*Escherichia coli* *Staphylococcus aureus* *Pseudomonas aeruginosa* *Streptococcus pyogenes*	Suspension	ND	Reduction in the concentration of 1 uM from 3 to 6 log_10_ CFU/mL. The greatest effect was in the first 30 s.
Yang et al. 2011 [15]	In vitro	WLBU2	100 s	652 nm	Temoporfin + WLBU2	*S. aureus* (methicillin resistant) *P. aeruginosa*	Suspension	3	Reduction by 100% for *S. aureus* (aPDT only and aPDT + peptide) and reduction by 2 log_10_ CFU/mL for *P. aeruginosa* (aPDT + peptide).
Liu et al. 2012 [16]	In vitro	WI13WF (YVLWKRKRKFCFI-amide)	2, 5, and 10 min	400 to 900 nm	Protoporphyrin IX	*E. coli* *Salmonella enteric* *Klebsiella pneumoniae*	Suspension	ND	Peptide and PS conjugate 99% lethal.
Dosseli et al. 2013 [17]	In vitro	Apidaecin	ND	600–750 nm 390–460 nm	Porphyrin	*E. coli* *S. aureus*	Suspension	ND	Reduction by 100% for *E. coli.*
Johnson et al. 2013 [18]	In vitro	(KLAKLAK)_2_	30 min	525 nm	(KLAKLAK)_2_ + Eosin Y	*Acinetobacter baumannii* *P. aeruginosa* *E. coli* *S. aureus* *Staphylococcus epidermidis*	Suspension	ND	Reduction by 99% for all microorganisms.
Dosseli et al. 2014 [19]	In vitro	Magainin Buforin	ND	390–460 nm	Porphyrin	*E. coli* *S. aureus* (methicillin resistant)	Suspension	ND	Reduction by 100% for all microorganisms.
Johnson et al. 2014 [20]	In vitro	(KLAKLAK)_2_	2 min 5 min 30 min	525 nm	(KLAKLAK)_2_ + Eosin Y	*E. coli* *S. aureus*	Suspension	3	Reduction by 50% for all microorganisms (2 min of irradiation). Reduction by 90% (5 min of irradiation). Reduction by 99.99% (30 min of irradiation).
Le guern et al. 2017 [21]	In vitro	Polymyxin B	20 h	420 nm	Porphyrin	*S. aureus* *E. coli* *P. aeruginosa*	Suspension	ND	Antibactericidal activity of the PS and peptide association on 3 strains.
De Freitas et al. 2018 [12]	In vitro	Aurein 1.2 (AU)	ND	660 nm	Methylene blue Chlorin e6	*S. aureus* *A. baumannii* *E. coli* *Enterococcus faecium*	Suspension	9	*S. aureus* reduction - MB ~ 1.0 log_10_ CFU/mL - MB + AU ~ 6.0 log_10_ CFU/mL - Ce6 and Ce6 + Au = total reduction *A. baumannii* reduction - MB ~ 1.0 log_10_ CFU/mL - MB + AU ~ 6.0 log_10_ CFU/mL - Ce6 and Ce6 + AU no significant results *E. coli* reduction - MB ~ 4.0 log_10_ CFU/mL - MB + AU ~ 4.0 log_10_ CFU/mL - Ce6 and Ce6 + AU no significant results *E. faecium* reduction - MB ~ 1.0 log_10_ CFU/mL - MB + AU ~ 3.0 log_10_ CFU/mL Ce6 ~ 1.0 log_10_ CFU/mL - Ce6 + AU = total reduction
Le guern et al. 2018 [22]	In vitro	Polymyxin B modified by lysine	20 h	420 nm	Porphyrin	*S. aureus* *E. coli* *P. aeruginosa*	Suspension	ND	Reduced antibacterial activity of polymyxin modified by lysine.
Nakonieczana et al. 2018 [23]	In vitro	CAMEL Pexiganan	668 s 1335 s 2668 s	514 nm	Rose-bengal (RB)	*P. aeruginosa*	Suspension	3	Reduction by 2.06 log_10_ CFU/mL for RB + CAM. Reduction by 6.00 log_10_ CFU/mL for RB + PEX.
Gao et al. 2019 [24]	In vitro	Magainin I	2 min 4 min 8 min	660 nm	Magainin I + Chlorin e6	*P. aeruginosa* *S. aureus* (methicillin resistant)	Biofilm	ND	*P. aeruginosa* 2 min (0.385 log_10_ CFU/mL reduction) 4 min (1.645 log_10_ CFU/mL reduction) 8 min (6.724 log_10_ CFU/mL reduction) *S. aureus* 2 min (0.922 log_10_ CFU/mL reduction) 4 min (3.796 log_10_ CFU/mL reduction) 8 min (6.586 log_10_ CFU/mL reduction)
De Freitas et al. 2019 [13]	In vitro	AU (GLFDIIKKIAESF-NH_2_) (AU)_2_K[(GLFDIIKKIAESF)_2_-k]	ND	664 nm	Methylene blue Chlorin e6	*Enterococcus faecalis* *S. aureus* *E. faecium*	Biofilm	9	Reducing the early biofilm stage - 95.5%—(Ce6-aPDT + (AU)_2_K) - 78%—Ce6-aPDT - 30%—MB-aPDT + AU - 20%—MB-aPDT - 30%—AU - 70%—(AU)_2_K)
Feese et al. 2019 [25	In vitro	Alkyne 1-Zn TMPYP	5, 15, and 30 min	400 to 700 nm	Porphyrin	*Mycobacterium smegmatis*	Suspension		Inactivation of 4 Log_10_ CFU/mL when associated with porphyrin and 1-Zn.
Zhang et al. 2019 [26]	In vitro	(KLAKLAK)_2_(KLA)	5 min (in vivo) 10 min (in vitro)	660 nm	PpIX PPK = PpIX + (KLAKLAK)_2_(KLA)	*S. aureus* *E. coli*	Suspension	ND	Inhibition rate *S. aureus* = 100% for both PS *E. coli* = 100% (PPK)/50% (PpIX)
Chu et al. 2021 [27]	In vitro	Bacitracin	5 and 30 min	610 nm	Phthalocyanine	*E. coli* *S. aureus*	Suspension	9	High phototoxicity of the Peptide with PS. The group without light 99% reduced.
Gao et al. 2021 [28]	In vitro/in vivo	PEGylated polypeptide	5 min	660 nm	PEGylated polypeptide + Chlorin e6	*P. aeruginosa*	Biofilm	ND	Total eradication of *P. aeruginosa* biofilms.
Judzewitsch et al. 2021 [29]	In vitro	ZnTTP-AC	30 min	Green-light irradiation	ZnTTP-AC	*S. aureus* *P. aeruginosa*	Suspension	3	4.5 log_10_ CFU/mL reduction for *S. aureus.* Total reduction for *P. aeruginosa*.
Qiu et al. 2021 [a] [30]	In vitro/in vivo	GKRWWKWWR-RPLGVRG	5 min	660 nm	GKRWWKWWR-RPLGVRG + Chlorin e6	*S. aureus* *E. coli*	Suspension	3	Total reduction for *S. aureus* 90% reduction for *E. coli*
Qiu et al. 2021 [b] [31]	In vitro/in vivo	GKRWWKWWRR	10 min 20 min 30 min	660 nm	GKRWWKWWRR + Chlorin e6 + AuNPs	*S. aureus* *E. coli*	Biofilm	3	*S. aureus* 10 min (~50% viability) 20 min (~20% viability) 30 min (~2.5% viability) *E. coli* 10 min (~60% viability) 20 min (~42.5% viability) 30 min (~10% viability)

ND: not documented; s: seconds; min: minutes: h: hour; PS: photosensitizer; ~: approximately; MB: methylene blue; RB: rose-bengal; Ce6: chlorin e6.

**Table 2:** Risk of bias assessment in the articles included, according to the OHAT criteria.

Studies/Questions	Was the Dose or Exposure Level Administered Adequately Randomized?	Was the Allocation to Study Groups Adequately Concealed?	Were the Experimental Conditions Identical Across Study Groups?	Were Research Personnel Blind to the Study Group During the Study?	Were the Outcome Data Complete without Attrition or Exclusion from the Analysis?	Is the Exposure Characterization Reliable?	Is the Outcome Assessment (Including Blinding of Assessors) Reliable?	Were There No Other Potential Threats to Internal Validity?
Bourré et al. 2010 [14]	++	++	++	--	++	++	--	--
Yang et al. 2011 [15]	++	++	++	--	++	++	--	--
Liu et al. 2012 [16]	++	++	++	--	++	++	--	--
Dosseli et al. 2013 [17]	++	++	++	--	--	++	--	--
Johnson et al. 2013 [18]	++	++	++	--	++	++	--	--
Dosseli et al. 2014 [19]	++	++	++	--	++	++	--	--
Johnson et al. 2014 [20]	++	++	++	--	++	++	--	--
Le Guern et al. 2017 [21]	++	++	++	--	++	++	--	--
De Freitas et al. 2018 [12]	++	++	++	--	++	++	--	--
Le Guern et al. 2018 [22]	++	++	++	--	++	++	--	--
Nakonieczana et al. 2018 [23]	++	++	++	--	++	++	--	--
Gao et al. 2019 [24]	++	++	++	--	++	++	--	--
De Freitas et al. 2019 [13]	++	++	++	--	++	++	--	--
Fesse et al. 2019 [25]	++	++	++	--	++	++	--	--
Zhang et al. 2019 [26]	++	++	++	--	++	++	--	--
Chu et al. 2021 [27]	++	++	++	--	++	++	--	--
Gao et al. 2021 [28]	++	++	++	--	++	++	--	--
Judzewitsch et al. 2021 [29]	++	++	++	--	++	++	--	--
Qiu et al. 2021a [30]	++	++	++	--	++	++	--	--
Qiu et al. 2021b [31]	++	++	++	--	++	++	--	--

++: direct evidence of positive finding; --: direct evidence of negative finding.
